# Domestic violence experienced by women with multiple sclerosis: a study from the North-East of Iran

**DOI:** 10.1186/s12905-022-01905-9

**Published:** 2022-07-31

**Authors:** Elham Manouchehri, Vahid Ghavami, Mona Larki, Morteza Saeidi, Robab Latifnejad Roudsari

**Affiliations:** 1 Department of Midwifery, Faculty of Nursing and Midwifery, Mashhad Medical Sciences, Islamic Azad University, Mashhad, Iran; 2 Department of Biostatistics, School of Health, Mashhad University of Medical Sciences, Mashhad, Iran; 3 Social Determinants of Health Research Center, Mashhad University of Medical Sciences, Mashhad, Iran; 4 Department of Midwifery, School of Nursing and Midwifery, Mashhad University of Medical Sciences, Mashhad, Iran; 5 Department of Neurology, School of Medicine, Mashhad University of Medical Sciences, Mashhad, Iran; 6 Nursing and Midwifery Care Research Center, Mashhad University of Medical Sciences, Mashhad, Iran

**Keywords:** Multiple sclerosis, Domestic violence, Psychological violence, Physical violence, Economic violence, Sexual violence, Iran

## Abstract

**Introduction:**

Violence against women is a significant health and legal problem and has been declared as a health priority by the World Health Organization. The most common type of violence against women is domestic violence, more prevalent against women with disabilities than other women. Multiple sclerosis (MS) is a debilitating neurological disease and has experienced sudden growth in Iran. This study aimed to investigate the prevalence of domestic violence and its various types (psychological, economic, physical, and sexual) experienced by women with MS.

**Methods:**

In this cross-sectional study, 275 married women with MS were selected using convenience sampling. After obtaining informed consent and reviewing the inclusion and exclusion criteria, the Domestic Violence against Women Questionnaire developed by Mohseni Tabrizi et al. was completed by the participants on a self-report basis. The results were analyzed using SPSS software version 16. To analyze data, statistical tests including chi-square and Fisher exact tests for univariate analysis and logistic regression, were employed.

**Results:**

The mean age of participants was 37.12 ± 8.48 years. Domestic violence in different forms of psychological, economic, physical, and sexual violence was present in 53.1%, 63%, 33.6%, and 20.4% of participants, respectively. Economic violence (33.8%) was the highest, and sexual violence (5.1%) was the lowest rate of severe violence among participants. There was a significant relationship between the overall rate of domestic violence and the variables including income (*P* = 0.013), spouse’s income (*P* = 0.001), participant’s job (*P* = 0.036) and participant’s education (*P* = 0.001). In logistic regression, the overall rate of domestic violence was higher in participants with education less than a diploma than in participants with a diploma (*P* = 0.014) and participants with a university education (*P* = 0.016).

**Conclusion:**

According to the results, providing opportunities such as promoting the social status of women, fulfilling the rights of women with disabilities and debilitating diseases such as MS in society is recommended. Additionally, educating men about the negative impact of domestic violence on the current and future status of the family seems necessary. Providing counseling facilities on various forms of violence, especially domestic violence, for women with MS, is also recommended.

## Introduction

Domestic violence, often known as domestic abuse or intimate partner violence (IPV), is any pattern of behavior aimed at establishing or maintaining dominance and authority over a partner. IPV is any kind of sexual, emotional, financial, or psychological harm or threat against another person. IPV is one of the most prevalent forms of violence against women worldwide [1].

From 2000 to 2018, the WHO and the UN Interagency Working Group on Violence Against Women analyzed data from 161 nations worldwide about violence. The results showed that one in three women around the world have been traumatized physically or sexually by a partner or someone else at a certain time in life [2].

Violence against women, in addition to creating physical, psychological, and social problems, also imposes a heavy financial burden on society [3]. Physical violence occurs in various ways and includes any antisocial behavior that harms a woman’s body [4]. Psychological and emotional violence are violent behaviors that damage a woman’s dignity, reputation, self-confidence, and personality and are challenging to assess and measure [6–8]. Sexual violence refers to any antisocial behavior that ranges from touching to rape and may occur in private marital and family life and may occur as a result of forced obedience to the husband or incest within the marital or family circle [8, 9]. Economic violence is such that in many parts of the world, women are unpaid workers and have to take care of their families for the rest of their lives and remain dependent on the male member of the family. It also includes depriving a woman of income from work or inheritance, locking her in the home, and not allowing her to work [10, 11].

One of the factors influencing the occurrence of domestic violence is a person’s health/illness [12]. Women with more disabilities have an increased risk of abuse and a greater likelihood of violence against them [13, 14]. According to a study conducted in New Zealand in 2021, people with at least one disability were more likely to report IPV throughout their lifetime than people without a disability, including women and men. This was true for all types of violence examined in the study (physical IPV, sexual IPV, psychological IPV, controlling behaviors, and economic IPV). In this study, Physical IPV was prevalent in 40.3% of women with at least one disability, 16.9% in sexual IPV, 60.3% in psychological IPV, 31.7% in controlling behaviors, and 24.7% in economic IPV [5]. Also, new research reveals that those with disabilities are at a greater risk of sexual violence from 7 to 40% [6–8]. In a meta-analysis study published in 2021, researchers examined the prevalence of sexual violence in adults with intellectual disabilities. The results showed that the prevalence of sexual abuse in adults with intellectual disabilities was 32.9% [9].

Although violence against women with handicaps or physical disabilities secretly imposes much burden on society, there is little scientific evidence regarding this population, as these people are ignored and abused [17].

Multiple sclerosis (MS) is one of the most common progressive and chronic disorders of the central nervous system that often causes chronic disability, and a severe decrease in physical activity occurs in these people [18].

MS occurs in people in their 20–50 s who are at their peak sexual activity and fertility [19, 20]. MS has significant economic and social effects and is the most common cause of non-traumatic disability in young adults [21]. According to the World Health Organization (2014), the prevalence of MS in North America and the United Kingdom is more than 100 per 100,000 people, Africa has the lowest prevalence of 5 per 100,000 people, Iran with 20–60 per 100,000 people has a moderate prevalence of MS [22, 23]. According to the Atlas of MS, last updated in 2020, Globally, females are twice as likely to have MS as males, and an increasing prevalence of MS reported across the Middle East in recent years [10].

The MS Association has estimated the number of patients with this disease in Iran at about 50,000 [25]. However, Iran has faced a sudden increase in the disease, especially in women [26], and every year, 5000 new cases are added to the number of patients in Iran [27]. MS affects not only the patient but also his/her family members, who often play a caring role. Divorce rates are six times higher in women with a chronic illness such as MS [28]. About 30% of people with MS need supportive care at home, and 80% of this care is provided by a spouse [29, 30]. The level of life satisfaction, intimacy, sexual self-efficacy, and marital relationship is reduced for the patient and caregiver spouse [11, 12].

Public health approaches to the primary prevention of domestic violence in women with disabilities focus on monitoring, identifying risk factors, and developing, evaluating, and disseminating interventions [13]. American Medical Association (AMA) recommends that all patients be screened for domestic violence [33]. In Iran, except for one pilot study in 2012, no research has been conducted to investigate domestic violence in women with MS. Understanding the causes of domestic violence leads to practical solutions. In presenting such solutions, it should be noted that an appropriate preventive policy should be based on two essential pillars, one is the prevention of domestic violence by men, and the other is the prevention of violence against women [35]. Women with MS exposed to domestic violence should be identified and receive supportive counseling. This study aimed to investigate the frequency of domestic violence in women with MS in Mashhad, Iran.

## Method

The present cross-sectional study was carried out on women diagnosed with MS and referred to the MS comprehensive clinic and MS society of Khorasan-Razavi in Mashhad, Iran, for treatment and medication from November 2020 to September 2021. The study participants were selected using the convenience sampling method. In this way, the researcher introduced the study to patients who were referred for specialist visits, treatment, and counseling. Patients were included in the study if they wished and after reviewing the inclusion and exclusion criteria. Sampling was continued until the sample size was sufficient. The inclusion criteria were being married, 18–50 years of age, approved diagnosis of MS by a neurologist based on the McDonald criteria [14], ability to understand and speak in Persian, being diagnosed with MS for a period of more than six months, and giving consent to participate in the study. The participants were excluded if they were addicted to drugs or alcohol, had another pre-existing major chronic illness (e.g., hypertension, epilepsy, diabetes, and cardiovascular disease) and/or psychiatric disorder, experienced a recent severe life crisis in the family, and had a recent history of surgery.

The researcher attended the Khorasan-Razavi MS Comprehensive Clinic and MS Society and invited all women with MS to participate in the research. Then, the research objectives were explained to every eligible participant. The questionnaires were filled by the participants in the presence of the researcher (EM), and if the participants needed more explanation about the questionnaire items, the researcher would give them more explanation.

According to the population of patients with MS registered in the MS comprehensive clinic and MS society of Khorasan-Razavi (3500 patients), and also considering that the number of female patients is three times more than male patients [15], the following formula was used to calculate the sample size.

$$n = \frac{{N \times \left( {Z_{{1 - {\raise0.7ex\hbox{$\alpha $} \!\mathord{\left/ {\vphantom {\alpha 2}}\right.\kern-\nulldelimiterspace} \!\lower0.7ex\hbox{$2$}}}} } \right)^{2} \times P\left( {1 - P} \right)}}{{N \times d^{2} + \left( {Z_{{1 - {\raise0.7ex\hbox{$\alpha $} \!\mathord{\left/ {\vphantom {\alpha 2}}\right.\kern-\nulldelimiterspace} \!\lower0.7ex\hbox{$2$}}}} } \right)^{2} \times P\left( {1 - P} \right)}}$$

Taking into account the first type error (α) of 0.05 and the prevalence ratio of domestic violence (*P*) of 50% (to reach the maximum sample size) and the accuracy (d) of 0.06, the sample size of 242 women was calculated. Assuming a 10% sample loss, the final sample size was 269 women with MS. Sampling continued up to 275 women.

### Violence against women questionnaire

Violence against women questionnaire was developed by Mohseni Tabrizi et al. [16], in which questions 1–10 include demographic information, and questions 11–21, 22–26, 27–32, and 33–36 include psychological violence, economic violence, physical violence, and sexual violence, respectively [16]. In the area of psychological violence, questions are asked about insults, ridicule, beatings, swearing, shouting, threatening to beat or threatening to throw objects, threats of divorce, ignoring the sensitivities and desires, insults to the participants' loved ones, not being allowed to leave the house, controlling all of the patient’s behaviors, and prohibitions from associating with others. In economic violence, the questions are about obsessive control of home expenses, putting the participant in financial trouble, not having access to family income and savings, forcing them to sell the property and, Mandatory receipt of participants' salary and income. In physical violence, the questions were about pushing, slapping, kicking, beating, and breaking or bruising limbs. In sexual violence, questions were asked about being forced to have an abortion, having forced sex, being forced to have unconventional behavior during sex or sexual behaviors in a way that was unpleasant for the participant.

The questions are graded based on Likert’s 5-point scale in which 4 = strongly agree; 3 = agree; 2 = don’t have any idea; 1 = disagree; 0 = strongly disagree. In the present study, the minimum and maximum scores of the questionnaire were 0 and 140, respectively. In each item, a score of 0 and 1 indicates low violence, a score of 2 indicates moderate violence, and a score of 3 and 4 indicates severe violence. The total score of domestic violence was categorized as follows: score less than 26 = low; score 26–52 = moderate, and score more than 52 = severe violence. In low domestic violence, the participant is not exposed to domestic violence, but in the moderate and high categories, the participant is under domestic violence.

Trying to evaluate the face reliability of the scale, Mohseni Tabrizi and his colleagues [16] used questions from previous research that were used by experts in a related study and were confirmed by the experts in the field of social sciences. In their research, the total Cronbach’s alpha coefficient was 0.83, which indicated acceptable reliability of the questions. In this study, the reliability of the questionnaire was measured again, and the total coefficient of Cronbach’s alpha was 0.8 concerning violence against women with MS.

In order to prepare the text in English, the variables of participant’s income and spouse’s income were reported in dollar. In this way, at the study, each dollar was equivalent to 4200 Tomans. Family support is definite to one’s perceptions of support from his/her entire family [17]. Responses were based on a 4-point scale of 1 (little or none), 2 (low), 3 (moderate), or 4 (strong).

WHO released ethical standards for researching domestic violence against women. These guidelines suggested eight recommendations: ensuring participant safety, designing methodologically-sound research to minimize misrepresentation, safeguarding the confidentiality of the participants, training and supporting the members of the study team, developing ways to minimize participants' experienced stress, educating field workers to refer and provide assistance in low infrastructure contexts, making sure results are appropriately interpreted and then used to develop policy [18]. The ethical considerations of this research included obtaining written and verbal informed consent and the right to withdraw from the research at any time. Throughout the research process, the research team remained faithful to the study’s main components and methodology. Participants in the study did not face any physical risks due to their participation. Participants were made aware of possible risks, such as the possibility of embarrassment as a result of study questions. Since the participants' identities were not recorded, everyone was given a unique number used for the analysis. In order to protect the privacy and confidentiality of participants' information, it was kept in a secure document and used only for research purposes. Due to a lack of expertise, the first author, who collected the data, was careful not to fall into the consultant role. Therefore, no advice was given to the women by the researcher. Instead, women who required counseling were referred to a psychologist at the center.

The study protocol was revised and approved by the Ethics Committee of Mashhad University of Medical Sciences. All participants signed an informed consent form before undergoing any assessment related to the study. Anonymity and confidentiality were guaranteed in all cases.

### Data analysis

All analyses were done using the statistical package for social sciences (SPSS), Version 21. At first, the data were analyzed descriptively, using mean and standard deviation for continuous variables and count and percent for categorical variables. In order to investigate the effect of demographic variables on each of the dimensions of domestic, the chi-square and Fisher exact tests were employed for univariate analyses. Then the variables with a *P*-value less than 0.1 in univariate analysis were entered into the multiple logistic regression. For fitting logistic regression, the dependent variable values were low violence (code = 0) vs. moderate or severe violence (code = 1). Model goodness of fit was evaluated through the Hosmer–Lemeshow test. The receiver operating characteristics (ROC) curves [and the corresponding area under the ROC curve (AUC)] were calculated to test for the discriminating performance of the model. All *P* values of less than 0.05 were considered statistically significant.

## Results

### Demographic characteristics

In the present study, 275 married women with MS were included in the study, and there was no sample loss. The mean age of participants was 37.12 ± 8.48 years. Of these, 87.3% (240) were younger than their spouses (Table 1). 37.1% of participants (102) had two children. The income of the majority of them (218, 79.2%) was less than 238 dollars per month, and the income of most of their spouses (150, 54.6%) was more than 714 dollars per month. Patients mainly were housewives (206, 74.9%), and most of their spouses were self-employed (140, 50.9%). The level of education of most participants and their spouses was 45.8% (126) and 47.6% (131) university education, respectively. Also, most of the participants (133, 48.4%) had strong family support.

**Table 1:** Characteristics of the study participants (N = 275)

Variables	Frequency	Percent
*Age difference*		
Less	240	87.3
More	18	6.5
Equal	17	6.2
*No. of children*		
0	40	14.5
1	86	31.3
2	102	37.1
More than 2	47	17.1
*Income (US dollars)*		
Below 238	183	66.5
238–476	35	12.7
476–714	17	6.2
714–942	21	7.6
More than 1190	19	6.9
*Husband income (US dollars)*		
Below 238	21	7.6
238–476	49	17.8
476–714	55	20
714–942	67	24.4
More than 1190	83	30.2
*Husband’s occupation*		
Employee	95	34.5
Laborer	31	11.3
Self-employee	140	50.9
Unemployed	9	3.3
*Occupation*		
Employee	69	25
Unemployed	206	75
*Educational level*		
Below Diploma	66	24
Diploma	83	30.2
University	126	45.8
*Husband’s educational level*		
Below Diploma	68	24.7
Diploma	76	27.6
University	131	47.6
*Family support level*		
No	40	14.5
Low	27	9.8
Moderate	74	26.9
Strong	133	48.4

### Frequency of domestic violence and its domains

Participants in the present study were exposed to domestic violence (moderate and severe) as follows: psychological violence (53.1%, 145), economic violence (63%, 172), physical violence (33.6%, 92) and sexual violence (20.4%, 56) (Table 2).

**Table 2:** Types and levels of domestic violence experienced by women with MS (N = 275)

Level of violence	Domestic violence dimensions								Total domestic violence	
	Psychological violence		Economic violence		Physical violence		Sexual violence			
	Frequency	Percent	Frequency	Percent	Frequency	Percent	Frequency	Percent	Frequency	Percent
Low	128	46.9	101	37	182	66.4	219	79.6	130	47.8
Moderate	104	38.1	79	28.9	78	28.5	31	11.3	127	46.7
sever	41	15	93	34.1	14	5.1	25	9.1	15	5.5

The highest rate of severe violence was in the domain of economic violence (33.8%, 93), and the lowest level of severe violence was in the domain of physical violence (5.1%, 14) (Table 2).

### Association between domains of domestic violence and independent variables

There was no significant association between age and the dimensions of violence except for economic violence so the mean age in women with economic violence was significantly less than women without economic violence (Table 3).

**Table 3:** Association between independent variables and dimensions of violence

Variables	Psychological violence			Economic violence			Physical violence			Sexual violence			Total domestic violence		
	− No (%) Mean ± sd	+ No (%) Mean ± sd	Results	− No (%) Mean ± sd	+ No (%) Mean ± sd	Results	− No (%) Mean ± sd	+ No (%) Mean ± sd	Results	− No (%) Mean ± sd	+ No (%) Mean ± sd	Results	− No (%) Mean ± sd	+ No (%) Mean ± sd	Results
Age	36 ± 8	38 ± 9	t = 1.45 *P* = 0.149	39 ± 8	36 ± 9	t = 2.39 *P* = 0.017	38 ± 8	36 ± 9	t = 1.32 *P* = 0.188	37 ± 9	38 ± 7	t = 0.49 *P* = 0.625	37 ± 8	37 ± 9	t = 0.41 *P* = 0.419
*Age difference*															
Less	110 (46.2)	128 (53.8)	$\chi^{2} =$ 2.6 *P* = 0.263	88 (37)	150 (63)	1.4 (0.5)	162 (67.8)	77 (32.2)	10.97 (0.004)	197 (82.1)	43 (17.9)	7.03 (0.03)	114 (48.1)	123 (51.9)	4.85 (0.09)
More	7 (38.9)	11 (61.1)		5 (27.8)	13 (72.2)		6 (33.3)	12 (66.7)		11 (61.1)	7 (38.9)		5 (27.8)	13 (72.2)	
Equal	11 (64.7)	6 (35.3)		8 (47.1)	9 (52.9)		14 (82.4)	3 (17.6)		11 (64.7)	6 (35.3)		11 (64.7)	6 (35.5)	
*No. of children*															
0	24 (60)	16 (40)	10.55 (0.014)	18 (45)	22 (55)	5.4 (0.145)	25 (62.5)	15 (37.5)	2.78 (0.427)	36 (90)	4 (10)	3.24 (0.357)	24 (60)	16 (40)	6.75 (0.08)
1	45 (52.9)	40 (47.1)		31 (36.5)	54 (63.5)		54 (63.5)	31 (36.5)		68 (79.1)	18 (20.9)		42 (49.4)	43 (50.6)	
2	46 (45.1)	56 (54.9)		41 (40.6)	60 (59.4)		74 (72.5)	28 (27.5)		79 (77.5)	23 (22.5)		49 (48.5)	52 (51.5)	
More than 2	13 (28.3)	33 (71.7)		11 (23.4)	36 (76.6)		29 (61.7)	18 (38.3)		36 (76.6)	11 (23.4)		15 (32.6)	31 (67.4)	
*Income (US dollar)*															
Below 238	80 (44)	102 (56)	6.54 (0.162)	62 (34.3)	119 (65.7)	9.25 (0.055)	118 (64.8)	64 (35.)	9.26 (0.055)	144 (78.7)	39 (21.3)	1.036 (0.911)	81 (44.8)	100 (55.2)	12.69 (0.013)
238–476	13 (38.2)	21 (61.8)		10 (28.6)	25 (71.4)		18 (51.4)	17 (48.6)		27 (77.1)	8 (22.9)		11 (32.4)	23 (67.6)	
476–714	10 (58.8)	7 (41.2)		6 (35.3)	11 (64.7)		14 (82.4)	3 (17.6)		14 (82.4)	3 (17.6)		10 (58.8)	7 (41.2)	
714–942	13 (61.9)	8 (38.1)		13 (61.9)	8 (38.1)		16 (76.2)	5 (23.8)		18 (85.7)	3 (14.3)		15 (71.4)	6 (28.6)	
More than 1190	12 (63.2)	7 (36.8)		10 (52.6)	9 (47.4)		16 (84.2)	3 (15.8)		16 (84.2)	3 (15.8)		13 (68.4)	6 (31.6)	
*Husband’s income*															
Below 238	10 (50)	10 (50)	12.57 (0.014)	5 (26.3)	14 (73.7)	32.1 (0.001)	9 (45)	11 (55)	35.15 (0.001)	16 (76.2)	5 (23.8)	2.32 (0.677)	9 (47.4)	10 (52.6)	24.98 (0.001)
238–476	15 (30.6)	34 (69.4)		7 (14.3)	42 (85.7)		23 (46.9)	26 (53.1)		41 (83.7)	8 (16.3)		12 (12.5)	37 (75.5)	
476–714	21 (38.2)	34 (61.8)		13 (23.6)	42 (76.4)		29 (52.7)	26 (47.3)		41 (74.5)	14 (25.5)		21 (38.2)	34 (61.8)	
714–942	33 (49.3)	34 (50.7)		28 (41.8)	39 (58.2)		48 (71.6)	19 (28.4)		52 (77.6)	15 (22.4)		33 (49.3)	34 (50.7)	
More than 1190	49 (59.8)	33 (40.2)		48 (57.8)	35 (42.2)		73 (88)	10 (12)		69 (83.1)	14 (16.9)		55 (67.1)	27 (32.9)	
*Husband’s occupation*															
Employee	43 (45.7)	51 (54.3)	4.72 (0.193)	41 (43.2)	54 (56.8)	7.42 (0.06)	75 (78.9)	20 (21.1)	18.01 (0.001)	79 (83.2)	16 (16.8)	6.93 (0.074)	47 (50)	47 (50)	10.28 (0.016)
Laborer	10 (32.3)	21 (67.7)		5 (16.1)	26 (83.9)		12 (38.7)	19 (61.3)		21 (67.7)	10 (32.3)		7 (22.6)	24 (77.4)	
Self–employee	72 (51.8)	67 (48.2)		52 (37.7)	86 (62.3)		90 (64.7)	49 (35.3)		114 (81.4)	26 (18.6)		73 (52.9)	65 (47.1)	
Unemployed	3 (33.3)	6 (66.7)		3 (33.3)	6 (66.7)		5 (55.6)	4 (44.4)		5 (55.6)	4 (44.4)		3 (33.3)	6 (66.7)	
*Occupation*															
Employee	40 (58.8)	28 (41.2)	5.182 (0.023)	32 (46.4)	37 (53.6)	3.486 (0.062)	50 (72.5)	19 (27.5)	1.51 (0.219)	57 (82.6)	12 (17.4)	0.502 (0.479)	40 (58.8)	28 (41.2)	4.42 (0.036)
Unemployed	88 (42.9)	117 (57.1)		69 (33.8)	135 (66.2)		132 (64.4)	73 (35.6)		162 (78.6)	44 (21.4)		90 (44.1)	114 (55.9)	
*Educational level*															
Below Diploma	15 (23.1)	50 (76.9)	24.03 (0.001)	9 (13.8)	56 (86.2)	19.87 (0.001)	30 (46.2)	35 (53.8)	23.56 (0.001)	48 (72.7)	18 (27.3)	5.53 (0.063)	14 (21.5)	51 (78.5)	25.58 (0.001)
Diploma	37 (45.1)	45 (54.9)		35 (42.2)	48 (57.8)		51 (61.4)	32 (38.6)		63 (75.9)	20 (24.1)		41 (50)	41 (50)	
University	76 (60.3)	50 (39.7)		57 (45.6)	68 (54.4)		101 (80.2)	25 (19.8)		108 (85.7)	18 (14.3)		75 (60)	50 (40)	
*Husband’s educational level*															
Below Diploma	23 (33.8)	45 (66.2)	6.22 (0.045)	18 (26.9)	49 (73.1)	4.195 (0.123)	36 (52.9)	32 (47.1)	8.995 (0.011)	47 (69.1)	21 (30.9)	7.435 (0.024)	25 (37.3)	42 (62.7)	4.082 (0.13)
Diploma	38 (50.7)	37 (49.3)		32 (42.7)	43 (57.3)		49 (65.3)	26 (34.7)		60 (78.9)	16 (21.1)		37 (49.3)	38 (50.7)	
University	67 (51.5)	63 (48.5)		51 (38.9)	80 (61.1)		97 (74)	34 (26)		112 (85.5)	19 (14.5)		68 (52.3)	62 (47.7)	
*Family support level*															
No	18 (45)	22 (55)	0.699 (0.873)	15 (38.5)	24 (61.5)	1.424 (0.7)	24 (60)	16 (40)	5.93 (0.115)	29 (72.5)	11 (27.5)	13.72 (0.003)	17 (43.6)	22 (56.4)	3.954 (0.266)
Low	11 (42.3)	15 (57.7)		7 (26.9)	19 (73.1)		13 (50)	13 (50)		18 (66.7)	9 (33.3)		8 (30.8)	18 (69.2)	
Moderate	37 (50.7)	36 (49.3)		27 (36.5)	47 (63.5)		48 (64.9)	26 (35.1)		53 (71.6)	21 (28.4)		36 (49.3)	37 (50.7)	
Strong	62 (46.6)	71 (53.4)		52 (39.1)	81 (60.9)		96 (72.2)	37 (27.8)		118 (88.7)	15 (11.3)		68 (51.1)	65 (48.9)	

The results of chi-square test showed that the psychological violence was higher in women with more than two children (*P* = 0.014), women with spouse’s income less than 1190 dollars (*P* = 0.014), un-employed women (*P* = 0.023), women with educational level lower than university (*P* = 0.001) and women whose spouse’s education was below diploma (*P* = 0.045).

The economic violence was significantly higher in women whose husband’s income were less than 1190 dollars (*P* = 0.001) and also women with lower education (*P* = 0.001).

Physical violence was significantly higher in families that the woman was older than her husband (*P* = 0.004); also, the physical violence was significantly higher in women with spouse’s income less than 476 dollars (*P* = 0.001), women whose spouse was laborer (*P* = 0.001), women with low education (*P* = 0.001) and women whose husband’s education was low (*P* = 0.011).

Sexual violence was higher in women older than their husband (*P* = 0.03), women with a lower spouse’s educational level (*P* = 0.024); also, the sexual violence was low in women with strong family support compared to other level of family support (*P* = 0.003).

The overall domestic violence was significantly lower in women with higher income (*P* = 0.013) and higher spouse’s income (*P* = 0.001). Also, the overall domestic violence was higher in unemployed women (*P* = 0.036) and women with laborer or unemployed spouse (*P* = 0.016). There was an inverse relationship between participant’s educational level and overall domestic violence (*P* = 0.001) (Table 3).

Variables with a *P*-value less than 0.1 in the last univariate analysis were included in multiple logistic regression. The results of logistic regression showed that the odds of psychological violence decreased in participants with a high level of education; so that the odds of the moderate or severe level of psychological violence in diploma and university education was 0.43 (95% CI 0.19–0.96) and 0.27 (95% CI 0.11–0.63) respectively, compared to women educated under diploma (Table 4).

**Table 4:** Results of logistic regressions (dependent variables were dimensions of violence: moderate or severe violence vs. low violence)

Variables	Psychological violence			Economic violence			Physical violence			Sexual violence			Total domestic violence		
	Odds ratio (OR)	Confidence interval	*P* value	Odds ratio (OR)	Confidence interval	*P* value	Odds ratio (OR)	Confidence interval	*P* value	Odds ratio (OR)	Confidence interval	*P* value	Odds ratio (OR)	Confidence interval	*P* value
Age	–	–	–	0.96	0.93–1	0.05	–	–	–	–	–	–	–	–	–
*Age difference*															
Less (Ref.)	–	–	–	–	–	–	1	–	–	1	–	–	1	–	–
More	–	–	–	–	–	–	4.21	1.37–12.91	0.012	5.81	1.8–18.78	0.003	1.95	0.58–6.5	0.280
Equal	–	–	–	–	–	–	0.49	0.11–2.12	0.337	4.71	1.48–15.02	0.009	0.54	0.17–1.73	0.298
*No. of children*															
0 (Ref.)	1	–	–	–	–	–	–	–	–	–	–	–	1	–	–
1	1.22	0.54–2.73	0.634	–	–	–	–	–	–	–	–	–	1.25	0.53–2.99	0.611
2	1.60	0. 71–3.58	0.257	–	–	–	–	–	–	–	–	–	1.34	0.57–3.16	0.504
More than 2	2.48	0. 92–6.68	0.071	–	–	–	–	–	–	–	–	–	1.7	0.61–4.76	0.314
*Income (US dollars)*															
Below 238 (Ref.)	–	–	–	1	–	–	1	–	–	–	–	–	1	–	–
238–476	–	–	–	1.39	0.54–3.58	0.497	1.92	0.81–4.52	0.138	–	–	–	2.17	0.85–5.56	0.108
476–714	–	–	–	0.98	0.29–3.37	0.978	0.45	0.11–1.86	0.272	–	–	–	0.89	0.27–2.97	0.847
714–942	–	–	–	0.61	0.19–2	0.412	1.63	0.46–5.72	0.449	–	–	–	0.51	0.15–1.79	0.296
More than 1190	–	–	–	0.87	0.25–3.04	0.823	0.91	0.21–3.97	0.904	–	–		0.57	0.15–2.1	0.396
*Husband’s income (US dollars)*															
Below 238 (Ref.)	1	–	–	1	–	–	1	–	–	–	–	–	1	–	–
238–476	2.04	0.63–6.65	0.238	1.71	0.39–7.5	0.475	0.56	0.16–1.95	0.364	–	–	–	3.08	0.79–12.04	0.106
476–714	1.92	0.61–6.05	0.264	0.95	0.23–3.9	0.942	0.5	0.14–1.76	0.276	–	–	–	1.9	0.5–7.21	0.343
714–942	1.50	0.46–4.86	0.500	0.63	0.17–2.38	0.497	0.28	0.08–0.98	0.047	–	–	–	1.63	0.46–6	0.459
More than 1190	1.05	0.32–3.37	0.94	0.34	0.09–1.32	0.12	0.1	0.03–0.39	0.001	–	–	–	0.81	0.22–3.02	0.750
*Husband’s occupation*															
Employee	–	–	–	1	–	–	1	–	–	1	–	–	1	–	–
Laborer	–	–	–	1.84	0.58–5.88	0.301	3.67	1.31–10.31	0.014	1.57	0.54–4.59	0.407	1.72	0.6–4.9	0.314
Self–employee	–	–	–	0.77	0.41–1.45	0.416	1.65	0.79–3.48	0.186	0.86	0.4–1.85	0.707	0.56	0.3–1.05	0.072
Unemployed	–	–	–	0.48	0.08–2.78	0.409	1.07	0.2–5.68	0.942	2.75	0.59–12.86	0.198	1.28	0.22–7.4	0.784
*Occupation*															
Unemployed (Ref.)	1	–	–	1	–	–	–	–	–	–	–	–	10.94	–	–
Employee	0.78	0.41–1.47	0.438	0.91	0.39–2.14	0.831	–	–	–	–	–	–	0.94	0.39–2.26	0.894
*Educational level*															
Below Diploma (Ref.)	–	–	–	1	–	–	1	–	–	1	–	–	1	–	–
Diploma	0.43	0.19–0.96	0.039	0.31	0.12–0.76	0.011	0.72	0.32–1.64	0.438	1.08	0.46–2.55	0.863	0.36	0.16–0.82	0.014
University	0.27	0.11–0.63	0.002	0.32	0.13–0.83	0.018	0.29	0.12–0.73	0.008	0.77	0.31–1.92	0.577	0.35	0.15–0.82	0.016
*Husband’s educational level*															
Below Diploma (Ref)	–	–	–	–	–	–	1	–	–	1	–	–	–	–	–
Diploma	1.03	0.46–2.34	0.936	–	–	–	1.6	0.68–3.78	0.283	0.75	0.31–1.86	0.539	–	–	–
University	1.31	0.58–2.97	0.519	–	–	–	1.62	0.69–3.8	0.271	0.51	0.2–1.27	0.146	–	–	–
*Family support level*															
No (Ref.)	–	–	–	–	–	–	–	–	–	1	–	–	–	–	–
Low	–	–	–	–	–	–	–	–	–	1.62	0.52–4.99	0.403	–	–	–
Moderate	–	–	–	–	–	–	–	–	–	1.45	0.56–3.7	0.443	–	–	–
High	–	–	–	–	–	–	–	–	–	0.33	0.12–0.89	0.028	–	–	–

Also, patients with a diploma and higher education had less chance to experience economic violence than patients with less than a diploma; so that the odds of economic violence in patients with an educational level of diploma and above was, respectively, 0.31 (95% CI 0.12–0.76) and 0.32 (95% CI 0.13–0.83) times of patients with an educational level less than diploma.

For patients who were older than their spouses, the odds of physical violence was 4.2 times (95% CI 1.37–12.91) of patients who were younger than their spouses. Also, in patients whose spouses earned more than 714 dollars per month, the odds of physical violence decreased. In patients whose spouse were workers, the chance of physical violence was 3.67 times (95% CI 1.31–10.31) of patients whose spouses were employees. Physical violence decreased in patients with higher education, so the odds of physical violence in patients with higher education was 0.29 times (95% CI 0.12–0.73) of patients with less than a diploma.

The odds of sexual violence in patients who were older than their spouses and patients who were the same age as their spouses was, respectively, 5.81 (95% CI 1.8–18.78) and 4.7 (95% CI 1.48–15.02) times of those who were younger than their husbands. Patients with strong family support had less chance, OR = 0.33 (95% CI 0.12–0.89) for sexual violence compared to patients without family support.

The overall chance of domestic violence was lower in patients with diplomas (OR = 0.36, 95% CI 0.16–0.82) and university education (OR = 0.35, 95% CI 0.15–0.82) compared to patients with lower education.

### Model evaluation

**Fig. 1:**
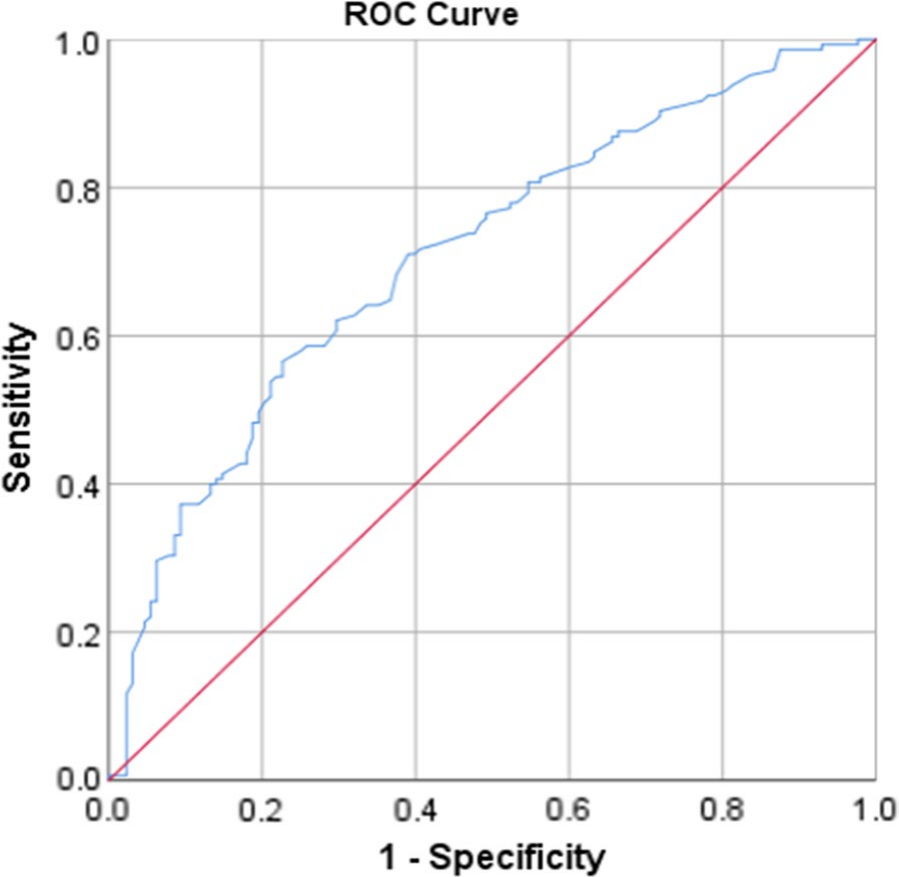
ROC curve of psychological violence

**Fig. 2:**
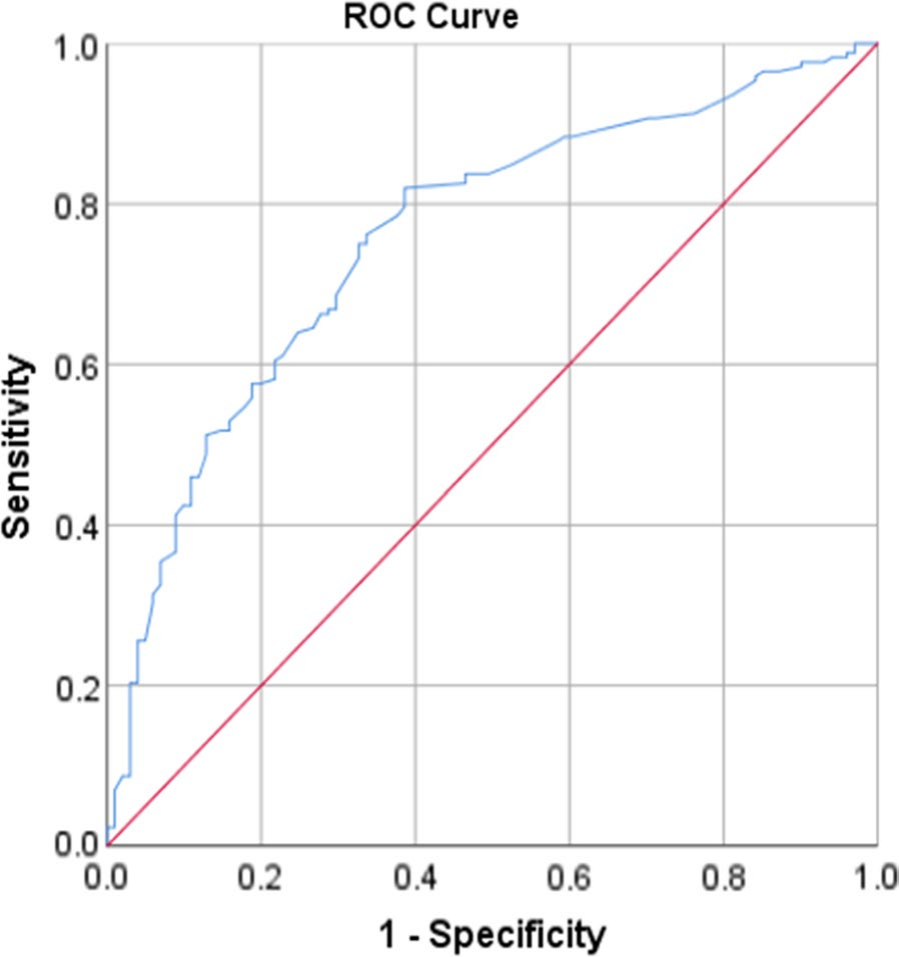
ROC curve of economical violence

**Fig. 3:**
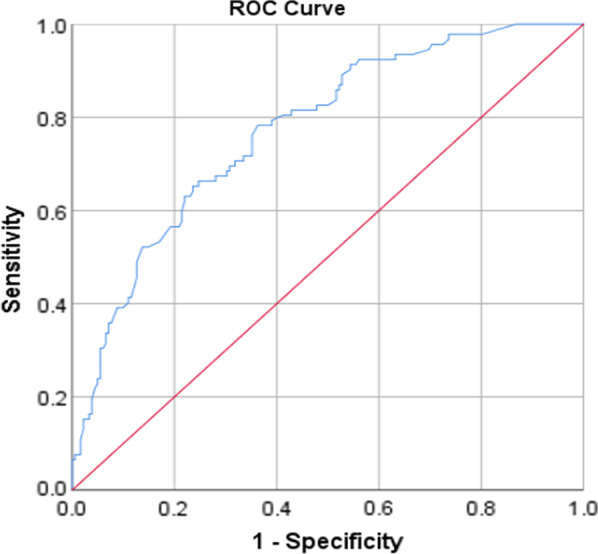
ROC curve of physical violence

**Fig. 4:**
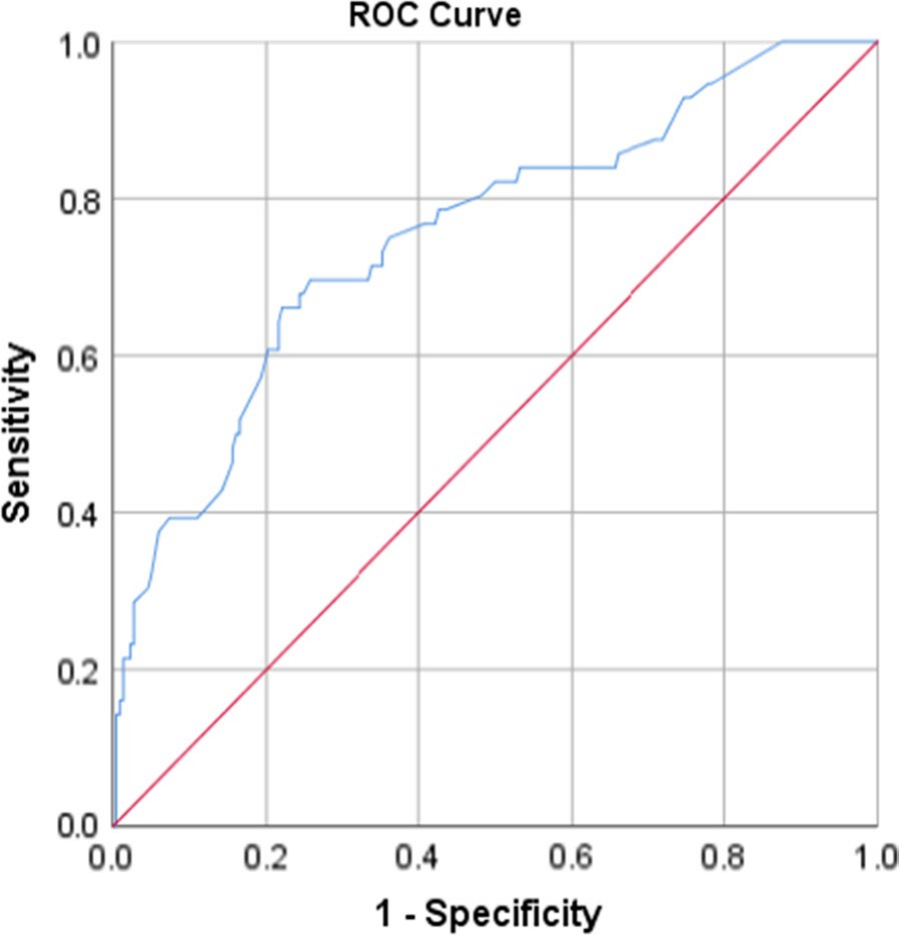
ROC curve of sexual violence

**Fig. 5:**
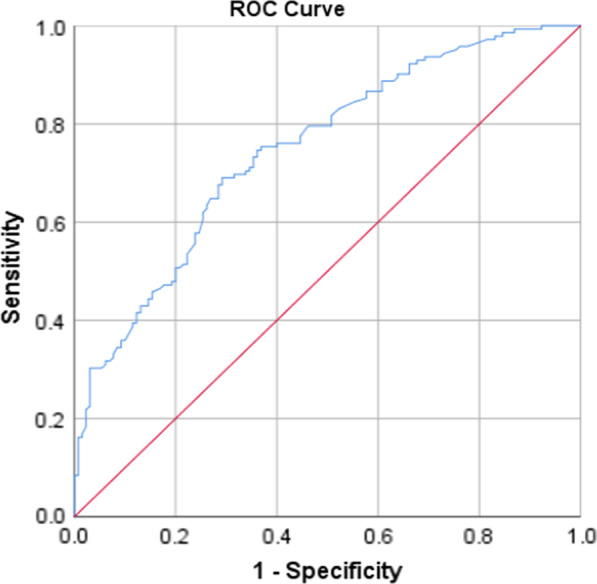
ROC curve of total violence

The goodness of fit of the logistic models was checked and confirmed using Hosmer and Lemeshow test (Table 5). Also, the AUC showed good discrimination of the models (Table 6, Figs. 1, 2, 3, 4 and 5).

**Table 5:** The results of the Hosmer–Lemeshow test

Dependent variable	results	Conclusion
Psychological violence	$\chi^{2} = 8.49, \;df = 8,\; P = 0.387$	Model was good
Economic violence	$\chi^{2} = 2.26,\; df = 8,\; P = 0.972$	Model was good
Physical violence	$\chi^{2} = 4.74, \;df = 8,\; P = 0.785$	Model was good
Sexual violence	$\chi^{2} = 11.29, \;df = 8,\; P = 0.186$	Model was good
Total violence	$\chi^{2} = 7.27, \;df = 8,\; P = 0.508$	Model was good

**Table 6:** The AUC results to test for discrimination in the models

Dependent variable	AUC [95% CI]	*P*
Psychological violence	0.70 [0.64–0.76]	< 0.001
Economic violence	0.75 [0.69–0.81]	< 0.001
Physical violence	0.79 [0.73–0.84]	< 0.001
Sexual violence	0.74 [0.66–0.82]	< 0.001
Total violence	0.75 [0.69–0.80]	< 0.001

## Discussion

This study aimed to investigate the prevalence of domestic violence and its different types (psychological, economic, physical, and sexual) in women with MS. The present study showed that women with MS face different types of violence from their husbands. These findings align with those of population-based research on people with disabilities and IPV conducted in the United States and Denmark [19, 20].

Among the types of violence experienced by women, economic violence and sexual violence were the most and less common violence experienced by women, respectively.

In evaluating global programs to address violence against girls and women with disabilities, more than a third of disabled women living in middle-income countries said they experienced physical and sexual violence in the past 12 months, and more than 40% said they experienced psychological violence from an intimate partner in the past year [21]. A pilot study about domestic violence in women with MS in Iran (2012) showed that the overall prevalence of physical, psychological, sexual, and any form of violence in the lifetime of women with MS were 17.8%, 38.4%, 6.8%, and 41.5% respectively. This study does not provide information on determining the extent of different types of violence [22]. According to a review study by Abraham [23], women with a disease or who do not have the appropriate level of general health due to physical disability are hesitant to have sex with their partner, which leads to sexual violence by them. They argued that most studies on sexual violence in women have shown that sick or newly recovered women are more vulnerable to domestic violence and that these findings are consistent with the findings of the present study [23]. There is additional evidence that women with disabilities in Ghana are subjected to a variety forms of violence, including social, verbal, physical, and sexual. From their perspective, this can be explained by the sociocultural background [24]. There was also a qualitative study by Opoku et al. [25] showing the majority of women with disabilities had been sexually abused, and that this was linked to other issues like poverty, social isolation, and unemployment [25].

The current study results showed that psychological violence is the second most common violence in women. (MS) is a chronic illness with significant psychosocial implications. Frequently, relationships with the patient’s spouse and other relatives are negatively affected. Therefore, programs are needed to deal with the psychosocial problems of this disease and to improve consequences for the person with MS and his/her intimate partner [26].

Dammeyer and Chapman [27] observed a significant difference in the type of violence or discrimination experienced. While men with disabilities are more likely to report physical violence, women are more likely to report sexual violence, humiliation, and discrimination [27]. Vulnerability to gender-based violence (GBV) is accelerated through the patriarchal authority the perpetrator has over the victim, little social support structures and poverty, disability, lack of potential or possibility to report on experiences of violence, economic or emotional dependence on the perpetrator [28].

In this study, a significant relationship was seen between the overall prevalence of domestic violence and the variables of participant’s income, spouse’s income, participant’s job, and participant’s education. In logistic regression, the overall rate of domestic violence was higher in participants with less education than a diploma than in participants with a diploma and university education than in a diploma. The overall rate of domestic violence was lower in patients with a diploma or higher than in patients with less than a diploma. In Giraldo’s study, there was also an inverse relationship between education and violence. They stated that the higher the level of education of individuals, the more they will understand and recognize the types of violence and naturally less exposed to it. Perhaps women with higher education have increased their awareness of their rights and reduced violence against them [29]. Heleta [30] examined the relationship between violence and educational level. He concluded that there was no significant relationship between increased education and reducing violence. This result was not consistent with the findings of the present study. He believed that the society’s culture, compared to education, has the most significant impact on the occurrence of all types of violence. He said that if violence is widespread in society, an increased level of education will take a long time to reduce violence [30].

In the current study, the relationship between economic violence and spouse’s income, participant’s occupation, and participant’s education was also significant. Considering the economic situation as a structural factor that causes violence in women, the result of the above study [30] confirms the correlation and significant relationship between the economic situation and violence against women with MS, perhaps because the cost of treatment itself is a stressor that leads to conflicts between couples. Women whose husbands are workers are more likely to experience physical violence. A study by Agaei on 1000 women between the ages of 15 and 64 who went to obstetrics and gynecology clinics found that the type of husband’s job was associated with domestic violence. Men working in government institutions were less likely to abuse their wives. Unlikely, in the study conducted by Aghaei, there was no correlation between the employment status of women and violence [31, 32]. In the present study, there was a relationship between women’s employment and violence that can be justified by women’s financial independence. The perceived powerlessness of a female with a disability and their perceived physical and financial dependency on perpetrators put them at more significant threat of violence inside the home and in their society [12]. According to a previous study, people with disabilities are more dependent on their partners for care, which might raise their chance of controlling behaviors and make it more challenging to leave partnerships [33].

The findings of the present study showed that patients with strong family support were less likely to have sexual violence than patients without family support. Social support is considered a resource that may act as a barrier against stressful situations such as discrimination and violence and may also help to improve one’s psychological well-being [34]. However, it is evident from the literature that favorable interactions with the social environment are beneficial to one’s mental health and well-being [35]. People with disabilities are deprived of their opportunities to enjoy the social life. This, along with exposure to violence and discrimination, may affect their health and well-being (60).

This study has provided an overview of the different forms of violence experienced by women with MS in Iran. Despite the critical contribution of the current study to the debate on violence, the results should be interpreted with caution due to the following limitations. The present study was a descriptive study of violence among women with MS in Iran. A longitudinal study that examines the predictive variables, the relationship between the variables, and the factors of occurrence of violence in this population, will be optimal. The researchers had challenges in recruiting samples that represented the community. One of the limitations of the present study is that although the sample size was appropriate because it was performed in the MS center in Mashhad, the results cannot be generalized to the whole situation of women with MS in Iran. Because Iran is a multicultural country. On the other hand, men as the primary decision-makers in the Iranian families and some cases, did not allow women to complete the questionnaires, making it impossible to assess the situation and depict violence against that group of women regarding violence against them.

Consistent with previous findings, intimate partners made up a majority of perpetrators of violence against individuals. However, as some disability scholars have demonstrated, IPV alone no longer reflects and represents the complexities of disabled women’s experiences of violence [36–39]. It is suggested that another study is needed to be designed to investigate the prevalence of non-domestic violence experienced by women with MS, and similar studies are conducted in other cities of Iran to provide an overview of the occurrence of violence in this population. It is also recommended that qualitative approaches be used to explore the experiences of violence in women with MS. Because the explorative and in-depth nature of qualitative studies could show why and how these women are vulnerable to violence in their daily lives. Except for a pilot study that was conducted in 2012 in the south of Iran, this is the first study conducted in Iran to investigate violence in women with MS. Therefore, the nature of the present study does not fill all the gaps in IPV prevalence data in women with MS. Nevertheless, existing data could pave the way for further research, service planning, efforts, and more comprehensive preventive responses to IPV in Iran. Therefore, designing support and care programs for women with MS can lead health care providers to achieve non-violent and peaceful psychosocial relationships with their husbands, improve quality of life and reduce the psychological and social burden of treatment processes.

## Conclusion

Women with MS experienced various forms of domestic violence throughout their lives. According to the results of the present study, providing opportunities such as the reconstruction of public culture in order to strengthen the status and human rights of women, promoting the social status and the rights of women with disabilities and debilitating diseases such as MS in society is needed. It is also recommended to educate men about the negative impacts of domestic violence on the current and future status of the family, and to expand counseling facilities on various types of violence, especially domestic violence, for women with MS. The expansion of MS health care and support services, including screening, early diagnosis, counseling, helplines, and management of domestic violence against these vulnerable populations, is also worth considering. It provides an opportunity for affected women to access appropriate health care and support services.

## Data Availability

The datasets used and/or analyzed during the current study are available from the corresponding author on reasonable request.

## Abbreviations

MS: Multiple sclerosis
WHO: World Health Organization
IPV: Intimate partner violence
AMA: American Medical Association
SPSS: Statistical package for social sciences
ROC: Receiver operating characteristics
AUC: Area under the ROC curve
GBV: Gender-based violence
